# agroString: Visibility and Provenance through a Private Blockchain Platform for Agricultural Dispense towards Consumers

**DOI:** 10.3390/s22218227

**Published:** 2022-10-27

**Authors:** Sukrutha L. T. Vangipuram, Saraju P. Mohanty, Elias Kougianos, Chittaranjan Ray

**Affiliations:** 1 Department of Computer Science and Engineering, University of North Texas, Denton, TX 76203, USA; 2 Department of Electrical Engineering, University of North Texas, Denton, TX 76203, USA; 3 Department of Civil and Environmental Engineering, University of Nebraska-Lincoln, Lincoln, NE 68588, USA

**Keywords:** smart agriculture, Internet of Agricultural Things (IoAT), Agriculture Cyber-Physical System (A-CPS), private blockchain, CorDapp

## Abstract

It is a known fact that large quantities of farm and meat products rot and are wasted if correct actions are not taken, which may lead to serious health issues if consumed. There is no proper system for tracking and communicating the status of the goods to their respective stakeholders in a secure way. Consumers have every right to know the quality of the products they consume. Using monitoring tools, such as the Internet of Agricultural Things (IoAT), and modern data protection techniques for storing and sharing, will help mitigate data integrity issues during the transmission of sensor records, increasing the data quality. The visibility state at the customer end is also improved, and they are aware of the agricultural product’s conditions throughout the real-time distribution process. In this paper, we developed and implemented a CorDapp application to manage the data for the supply chain, called “agroString”. We collected the temperature and humidity data using IoAT-Edge devices and various datasets from multiple sources. We then sent those readings to the CorDapp agroString and successfully shared them among the relevant parties. With the help of a Corda private blockchain, we attempted to increase data integrity, trust, visibility, provenance, and quality at each logistic step, while decreasing blockchain and central system limitations.

## 1. Introduction

**Figure 1:**
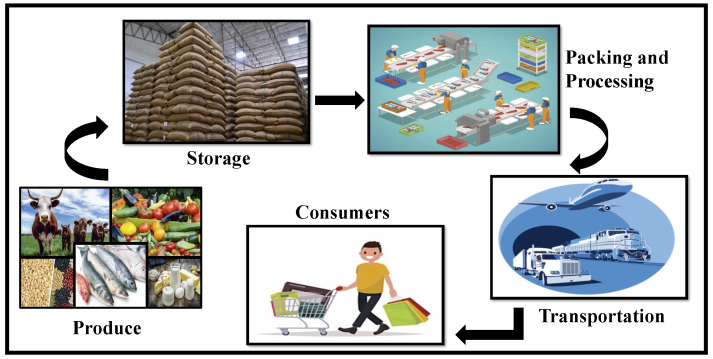
Stages in agricultural product distribution towards the consumers.

Agriculture plays a vital role in food production and is one of the primary sources of the farmers’ daily livelihood. Food is the means of nutrition for the world population and is essential for increasing a country’s economic status. Agriculture is comprised of farming crops, livestock, poultry, beekeeping, forestry, and sericulture. With the population projected to grow approximately from 6.9 billion people to 9.3 billion by 2050 [1], the demand for food production is expected to increase at a high rate. According to the U.S. Department of Agriculture, 40% of the produce is wasted and lost from the food supply every year [2] due to different factors, out of which distribution of farm produce contributes the highest percentage. As a result, more robust technological solutions are required to secure agricultural produce and transport fresh goods to the consumers, reducing wastage and providing trust in the process through visibility and provenance. The different trading options initiated, and transports instigated, have increased the accessibility of agricultural produce to even remote locations and long distances. Although shipping facilities have taken key roles in food supplies, factors like trust, quality, and time delivery impact the agricultural supply chain, consequently increasing the cost of the product for the consumers directly [3]. Figure 1 shows different stages in agricultural produce distribution from producer to the end consumers.

**Figure 2:**
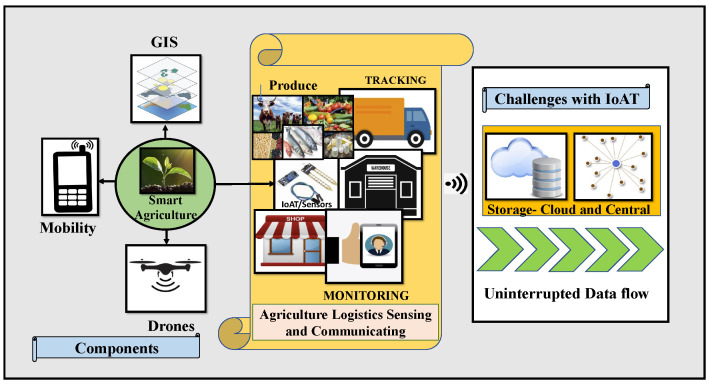
Components of smart agriculture and challenges of the IoAT.

Smart agriculture is one of the application fields of intelligent systems whose goal is to provide solutions for business processes using the IoAT (Internet of Agricultural Things). Advanced industries are using the IoAT and web-based solutions for incorporating modern techniques for data collection and the distribution of agricultural produce, along with implementing automation with security. Still, these approaches have limitations when bringing heterogeneous data into a unified system, raising privacy challenges and futile access control mechanisms [4]. Smart “things” employing a single point database system to store the statistics are having latency problems and Internet discontinuity issues for data flow and face possible attacks on the useful information [5]. Figure 2 demonstrates the components utilized in Smart Agriculture and some of the challenges of the IoAT in the agricultural product supply flow [4].

Technologies like the IoAT, AI/ML, Robotics Systems, and the blockchain play an essential role in making agriculture smart. Some of the relevant research works in smart agriculture include studying the health of the crops for disease detection [6], monitoring the growth of crops [7], crop damage estimation-eCrop, aerial vehicles for detecting wildfires [8], livestock tracking [9], and distributed ledger technologies for securing data sent in real-time [10,11].

A blockchain is a distributed ledger that works on the principle of decentralization, allowing multiple parties to have a consistent view of the data transactions without a single authority or security embedded around it through cryptographic calculations. Participating nodes with copies of the written data can vote and agree to a single decision and make the information valid by employing a consensus mechanism. Writing data onto the public blockchain can require rewards and computing power, consequently limiting the volume of data transfers and resulting in higher energy consumption. From the public domain cryptocurrencies, such as Bitcoin and Ethereum, the blockchain has evolved to private Enterprise Blockchain (EBC), which is more beneficial for industrial data exchange among relevant parties by mutual agreement.

The following is the order we present the current paper: Section 2 elaborates on some of the main issues that are present in the agricultural product distribution. Section 3 discusses the novelty through the usage of CorDapp application for the current paper. Section 4 examines previous works in the agricultural domain and compares them with the current agroString application. Section 5 gives the novel architecture for our current agroString application. The presentation of the algorithms that are used in agroString is given in Section 6. Section 7 and Section 8 provide implementation details and the results for the CorDapp application used in the current paper. Finally, conclusions and suggestions for future improvements are discussed in Section 9.

## 2. Concerns and Challenges of Agricultural Production Distribution

**Figure 3:**
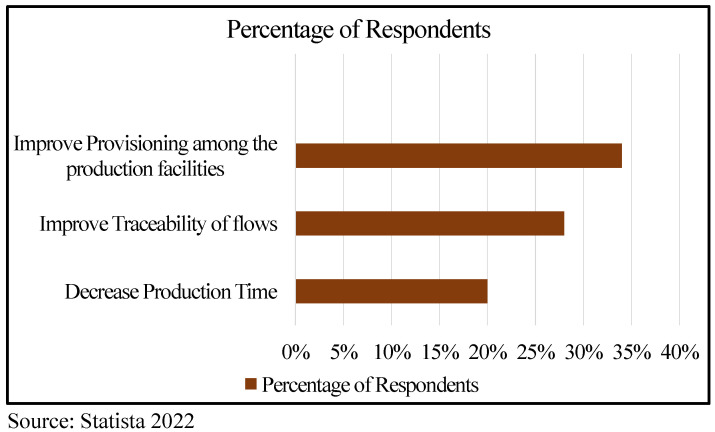
Statistics of consumer survey on challenges and issues faced by produce distribution.

A survey was conducted to elucidate the challenges and issues faced by agricultural produce distribution (Figure 3). It shows that 34% of the people surveyed were focused on improving provisions among the production facilities, 28 % were focused on improving traceability of the agricultural produce, and 20% were focused on decreasing the production time [12]. Here, we discuss some of the problems that can stem during agricultural production, processing, packaging, and distribution and due to the farmer’s knowledge of technology [13].

**Figure 4:**
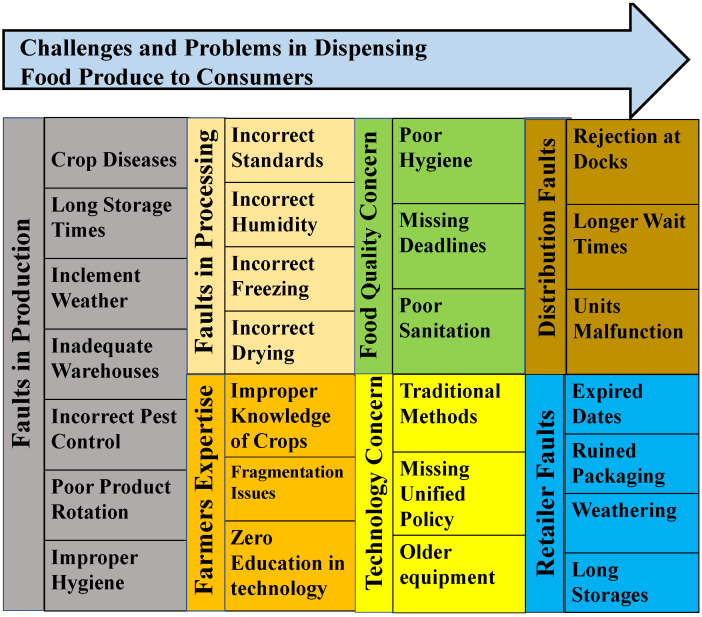
Concerns and challenges of agricultural production distribution.

Food quality is an essential concern in a supply chain because it is directly related to the well-being of individuals. The quantity of high-quality agricultural products available to the consumer can be unexpectedly reduced for various reasons at different stages of the supply chain, as shown in Figure 4. Circumstances for food quality degradation can occur due to unhealthy sanitation, unhygienic conditions, and missing deadlines between the supply chain participants. In food production, due to disease and inclement weather conditions, the crops can go unharvested and left in place to waste. Cosmetic imperfections of the produce can occur either during or after the harvest. Inefficient and long storage times after the harvest lead to production cost diminution. Storage issues can cause a lack of proper hygienic places for keeping the product nutritious and safe to consume. Due to inadequate warehousing facilities, wrong temperature, humidity, incorrect pest control procedures, and poor product rotations, the food quality can be degraded, making farmers face difficulties in getting reasonable prices from purchasers [14]. During the processing of the products, freezing, drying, and slicing are standard measures for making any frozen foods or produce. If the procedure cannot maintain correct temperatures during manufacturing, it can lead to the spoilage of food.

As farmers play a significant role in producing a healthy crop, their knowledge of technology usage on the field and active communication with food distributors will help in properly loading and transporting the produce toward end-users. However, inadequate knowledge and expertise in the current technologies have resulted in an increase in fragmenting issues from producers to intermediaries to consumers. With limited and inefficient technologies, the communication between the farmer and the intermediaries is complicated and uncertain. Using traditional methods and older equipment by the producers, it is hard to bring them under a single policy for the post-harvest phases in real-time. Longer waiting times, or rejection at the loading docks, malfunctioning of the refrigerating units, and accidents during truck transports can lead to an expiration of the produce shelf life. The long delays can result in ruined packaging and damages through weathering, which can lead to depreciated value and quality of the agricultural goods near the retail shops before they become available to consumers. A few practices, such as on-time delivery, maintaining hygienic requirements, and good sanitation standards, help increase the food’s quality.

## 3. Novel Contributions

### 3.1. Why Blockchain in Smart Agriculture?

**Figure 5:**
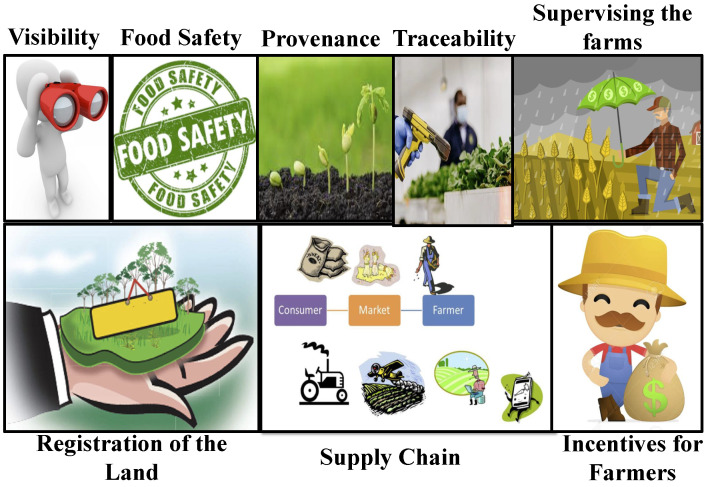
Blockchain use cases in smart agriculture.

The blockchain in smart farming plays an essential role in increasing trust in the data collected from inventories and farms. With the blockchain, communication and provenance of the goods can be provided to the consumers to verify product hygiene. This helps in avoiding food waste and keeps the product fresh until it reaches the end-users. With the blockchain introduced in agriculture, the information of the carriers, registering of the stakeholders and logistics involved, data regarding condition, price, and quality of the goods can be made visible, while also maintaining strict documentation for the products. With the blockchain, pricing imbalances can be avoided and the farmers have the full right to the price decisions on their supplies. Blockchain cryptocurrency can also be delivered as incentive to those participants/farmers who have optimally used resources and practiced eco-friendly options for growing crops [15]. Figure 5 provides some of the use cases of the blockchain in smart agriculture.

**Figure 6:**
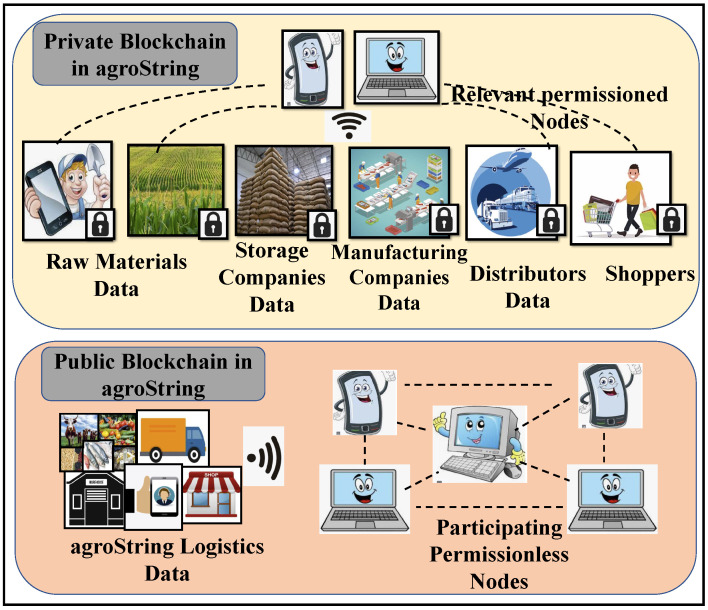
Comparison between private and public blockchains in supply chain.

Figure 6 shows the differences between private and public blockchains in the supply chain. With the use of a permissioned private enterprise blockchain distributed platform, the IoT limitations of data storage and security can be addressed to produce immutability, interoperability, and access control through smart contracts. Due to excessive resource consumption, traditional blockchain consensus mechanisms cannot be utilized with smart things. In the current paper, we have attempted to develop and implement a blockchain Corda-based data sharing framework for conventional agricultural produce distribution to the consumers by collecting the condition of the products at every stage with the help of the IoT.

### 3.2. Problems Addressed in the Current Paper

- Storage of data from the IoAT in central and cloud systems.
- Excessive transaction fees and mining time issues related to a public blockchain.
- Sharing of data to all the nodes that are participating.

### 3.3. Solutions Proposed in the Current Paper

- Evade centralized storage and implement decentralized storing and sharing.
- Use a private blockchain, also referred to as a permissioned blockchain.
- Propose a novel architecture for traceability and provenance in agroString.
- Reduced mining times.

### 3.4. Novelty and Significance of the Proposed Solutions

- Novel approach of distributed ledger technology for zero transaction fees (no cryptocurrency).
- Consistency and standards in communication between relevant parties with DeFi (Decentralized Finance) methodology for sharing the data transactions within permissioned peers with no intermediaries and within organization firewalls.
- A novel CorDapp private blockchain application that can be programmed.

## 4. Prior Related Work

In recent years, research and development work had been held between agricultural farmers and researchers. Various studies are being implemented to enhance mechanical and automatic methods for fusing information processing concepts and other fields.

With the help of Radio Frequency Identification (RFID), traceability is implemented for the fish supply chain in [16]. An architecture for live fish processing is designed and proposed for small enterprises. Each live fish has an RFID tag placed that connects it to logistic centers and retailers in order to provide the individual identities of the fish to the consumers. Sensors collect information during farming and transport. This work also makes use of web-based design for ease of use for both farmers and consumers.

To reduce the cost and load times of blockchain, a 2021 study [11] sent groundwater nitrate contamination data towards distributed storage in the Interplanetary File System (IPFS) and blockchain (Ethereum) for implementing dual hashing security and access control strategies.

In [17], an agro-food supply chain is proposed with the help of RFID for traceability with public blockchain technology. The distributed storage platform embedded in the system brings traceability with trusted information for the chain that enhances food safety throughout the supply process.

An IoT sensor was used for the tracking of cows in [18]. The paper presents a LoRaWAN architecture for communicating between long ranges and analyzes cattle tracking through a high-level system architecture. The protocols and application of the system are also further designed and developed.

For traceability in agriculture supply chain management, researchers [19] have executed a farm-to-fork blockchain. Both Ethereum (public blockchain) and Hyperledger Sawtooth (private blockchain) methods are implemented here. The performance is compared between both deployments for CPU and network usage, and their pros and cons are summarized.

Others [20] provide a theoretical representation of the whole supply chain network, starting from the provider to the consumer with the blockchain. The presented methodology attains traceability along with improved security, immutability, and faster transactions. The main objective is to provide transparency and accessibility to all the users of the supply network chain.

A traceability system [21] is designed with a dual storage structure, a blockchain as on-chain storage, and a traditional database for off-chain storage in order to decrease the load and cost and increase the ability to share traceability data safely.

The work in [22] uses the IoT to collect data and extends the application by embedding an Ethereum public blockchain to share data collected from the logistic stages securely. Through this, the system’s performance is increased, securely transmitting the data and growing trust in data collected from relevant supply chain parties. Using drones in supply chain management is a recent IoAT progress in smart agriculture. Drones have many limitations when it comes to real-time usage, such as energy-draining, routing problems, and disposal effects [23]. The work in [24] introduces a system with blockchain data sharing to meet the issues of drones and proposes a mixed-integer programming model to formulate the intended problem and solve the problem with a tailored branch-and-price algorithm.

A plethora of research has been implemented for secured data flow for enterprise systems and health care using the blockchain, smart things, and similar techniques. From the IoT viewpoint, the blockchain has been implemented for enhancing data security and integrity through documenting health metrics in healthcare [25]. Contemplating the above literature, we have tried to develop a blockchain for the agri-dispense-data sharing system agroString between appropriate stakeholders. We have used IoAT-Edge device data and various records (Table 2, discussed in detail in Section 6) for averting manual recording, and we believe that using EBC technology and various sensors will improve real-time data sharing and augment security. The key point for Corda is that it can be employed to share basic text and numerical data between the relevant parties only. Table 1 shows a comparison of various agricultural data management applications with the current agro-String data management.

## 5. Architecture of the Proposed agroString

With the increasing demand and worldwide sourcing for food distribution for the global population, the safety and quality of agricultural produce has become a severe challenge. Some of the consequences of poor distribution can be related directly to the increase in product life-cycle cost, spoilage, and waste, along with bad efficiency. Here, efficiency is measured using the fraction of both transportation (Tn) and distribution (Dn). The capacity at which Tn and Dn can be achieved is calculated to obtain a snapshot of the food quality at a given time. The efficiency estimates for trucks and transports fall in between the range of 10 to 20%, and food spoilage and waste that are caused due to untimely delivery is approximately 12% [26]. More than 48 million people in the United states get sick due to food borne illness at least once every year [26].

To deal with and address some of the challenges of quality and timely delivery, the logistics need some technological facets that can provide tracking of the whole life cycle from providers and producers to costumers. The intermediaries include storage/warehousing, processing/packaging, transportation, and retailers/shops. The architecture shows the trail based on IoAT and EBC between the trusted parties. The flow of the data would be linear and only among those relevant.

### 5.1. Internet of Agriculture Things—Sensors and Networks for Quality Tracking and Communication

In the proposed architecture, the logistic stages that use the techniques for checking the temperature and humidity controls for agricultural goods are fixed and would require a more refined practical approach in real-time. By using the IoAT, the current condition of the produce and transmitting times of the product can be monitored. With the help of faster network connections, the data can be communicated in real-time and broadcast to the logistic strings [26].

For the warehouse and logistics phases, intelligent devices can be implanted to measure the quality. Every phase included in the food supply string can act as a communicating point, and the recorded flow of information would be provided to the retailers and consumers at the end for quality verification. We can achieve product tracking through sensing and communicating with the help of intelligent things. Continuously sensing temperature, humidity, and bacterial content accumulated on the produce [27] can reduce food deterioration with efficient timely remedial actions.

### 5.2. Private Blockchain—Achieving Access Control/Privacy/Trust in agroString

The blockchain is one of the fastest evolving technologies for secured data exchange and business operations. One of the leading platforms in this developing area is Corda. It takes the properties of a public blockchain, such as bitcoin and Ethereum, where anyone can initiate transactions, and fulfills the enterprise’s requirements by inserting privacy and identity. Corda implements distributed, decentralized, permissioned, and open source Smart Contracts (SC). With the help of SC, access control, data integrity, privacy, and immutability are achieved [28].

Public and private blockchains are being used in supply chain applications to secure data transmitted between stakeholders. Blockchains have blocks connected in a chain pattern and utilize mining strategies for sending and receiving data. Corda, on the other hand, does not function with these approaches. In the present architecture, we demonstrate Corda-based private blockchain for receiving quality data of agricultural goods in the form of transactions near the communicating points. Corda transactions share the information between the connected logistic parties and charge zero transaction fees. Each transaction is provided with a tag containing secret codes belonging to the data that needs to be exchanged and shared between the relevant groups [28].

When digital files are reused, copied, and pasted several times, the value of the document rapidly becomes null, leading to double spending problems. Corda mitigates and prevents this issue by using a notary, deciding to sign if no problems arise and to not sign the transaction if double-spending occurs.

### 5.3. Consensus Mechanism—Corda Private Blockchain

Corda private blockchain does not use proof-of-work or proof-of-stake consensus mechanisms but still acts like a blockchain when adding a transaction to a ledger. The consensus is achieved by proving that the transaction is both valid and unique. Every transaction is a combination of states. Each state that is consumed is called input, and each state that is produced is referred to as output. When a state is consumed, it is like a money note being transferred to another party and will be marked as spent; each of these states carries a unique identifier, which is a combination of both Merkle hash and index number. For validity consensus, Corda checks that every transaction generated with input and output state is accepted by the smart contract of every input and output state and obtains all the required signatures for the transactions. This process is called walking the chain. The second is the uniqueness consensus, in which a notary checks for a node that has not used the same input state for multiple transactions. The transactions must achieve both validity and uniqueness consensus to be committed to the ledger in the Corda private blockchain.

### 5.4. Architecture

**Figure 7:**
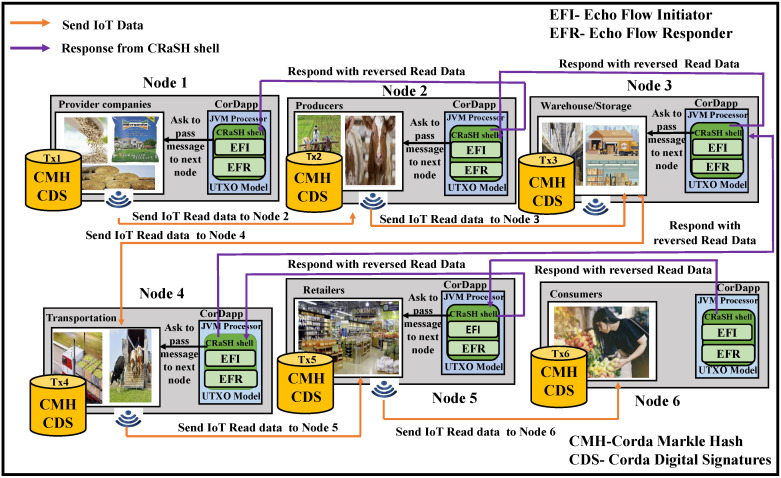
Proposed agroString architecture with IoAT and CorDapp.

Every node is unique and maintained by different institutes or companies belonging to agricultural stakeholders. In the architectural design presented in Figure 7, each logistic stage represents an independent node managed by their respective data centers connected via the Internet. The IoT devices are connected to each of these agricultural string logistics embedded with the Corda Shell application blockchain. The starting node contains an initiating flow that is given from the command-line CRaSH shell, which is a JVM-based instance. This flow helps start the entire echo process by sending the collected information from the IoAT to the recipient’s node. The second node waits for the incoming data to send an acknowledgment by means of a responder flow. The shell acts as a bridge between the Corda container and its services within the JVM. After initiator and responder flows are written, they are packed, organized, and compiled in respective JAR files. These JAR files can be shared among the nodes that are germane. The communication between each logistic node is a similar process using Corda flows for tracking, saving, and forwarding immutable data towards consumers in the end.

## 6. The Proposed Algorithms

Each logistic node represents the stakeholder present between the producer and consumer in the agricultural produce distribution. Each of these nodes sends requests for uploading IoAT (Datasets Table 2) data in real-time for signing and encrypting data. An elliptic curve mathematical function is used to generate private and public keys in these nodes. The uploaded data signifies a transaction that gets hashed and signed through the digital signatures exchanged among the nodes. Hash checks are performed to check for the integrity of the files. If the hashes are similar, the IoAT data gets signed successfully to start the flow of the data in between relevant nodes. The complete process for uploading and encrypting IoAT Data in CorDapp is given in Algorithm 1. Once the data gets uploaded to the node, the CorDapp encrypts using digital signatures distributed through the Certificate Authority (CA). The nodes participating exchange the certificates for sharing private and public keys to establish data legitimacy, valid proof of origin, and to make sure correct recipients obtain the data. As soon as the relevant node gets the encrypted data, the decryption process is initialized to check for the validity of the received data. Each node generates a public and private node in the decryption process, respectively. With the help of the Merkle tree, the encrypted transactions present in the logistic nodes are retrieved. Each of these transaction signatures are compared to match the sender and receiver signatures for starting decryption. The CorDapp creates the flow if the decrypted transaction is similar to the uploaded transaction. A detailed flow of the decryption and retrieving of the file is given in Algorithm 2.
**Algorithm 1** Uploading and Encrypting IoAT Data in CorDapp.
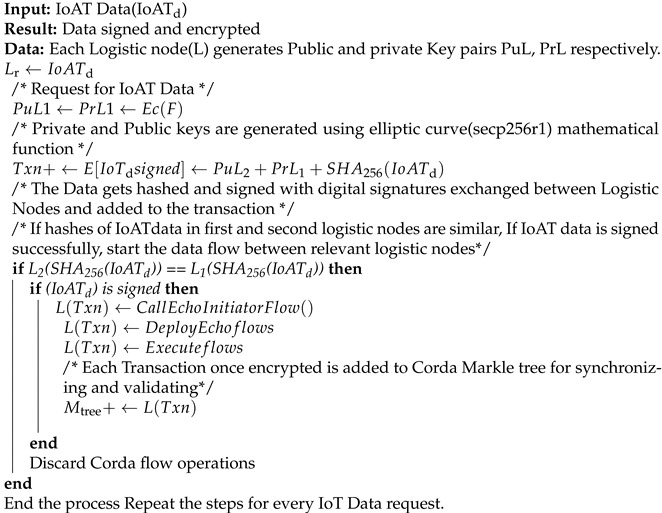

**Algorithm 2** Accessing and Decrypting IoT Data.
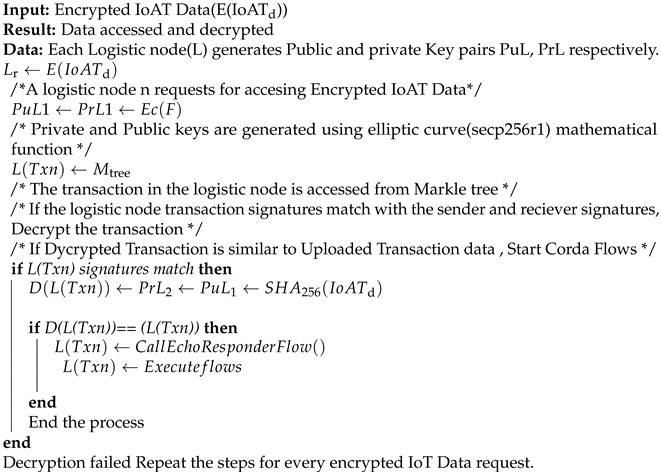


## 7. Implementation of the Proposed Blockchain

For the current system, we have chosen Corda with java objects. The main point of using this system is that the data shared is only between relevant parties. Each node will send the transaction data along with tags of secret codes to make the sharing safer. With this secure hashing approach, each transaction avoids alterations to the data in later stages. The records are written in the format of Unspent Transaction Objects (UTXO) [28], which is the basic model of bitcoin. In order to represent states in transactions, we have written them as plain java objects. A notary’s signature is embedded whenever there is a transaction. We have used JVM bytecode to define the structure and parameters of the state for more flexibility. Unlike other blockchain approaches, where transactions are broadcast to outsiders, Corda does not, because a notary prevents airing and double-spending and gives uniqueness to the transaction, as discussed in the mechanism of consensus in Corda in Section 5. The following implementation has been conducted in two phases.

### 7.1. Sensor Data

**Figure 8:**
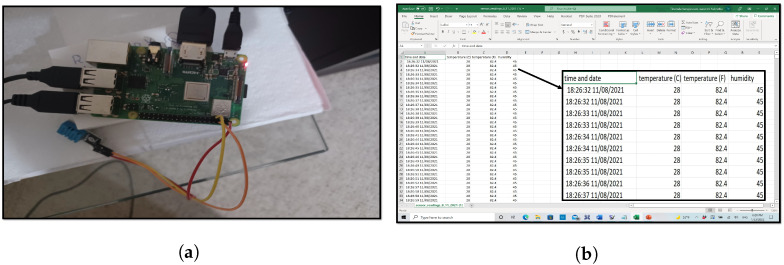
Sensor data. (**a**) IoT-Edge device. (**b**) IoT-Edge device data in CSV format.

To perform phase one, we have collected temperature and humidity data using a sample IoAT-Edge device (DHT11 sensor + Raspberry Pi 3 Model B+) to obtain approximately 60,000 records, as shown in Figure 8. The gathered statistics were sent to the CorDapp application as a .zip file. Along with the real-time IoAT-Edge data, we also tested our agroString application with different datasets that are discussed in detail in Section 8.1.

### 7.2. CorDapp agroString Application

In phase two, we cloned an already defined starter template to git for building our CorDapp. The call method in the Initiatorflow() was used to write the application logic in java. With the help of @InitiatedBy annotation in the ResponderFlow(), we associated it with the InitiatorFlow(). The Initiatorflow call method has different services for executing the logic. One of the identity services in this call method provides an address book from which we can retrieve the identity of the recipient and locate it. In our application, the message carried is the sensor data in the form of .csv converted to .zip. For matching the recipient’s name, we used the partiesFromName method in the service to the recipient string. Once the recipient was matched, we received the list of party, and,  using the send() method, we forwarded the sensor data to the following logistics recipient nodes.

To deploy and compile our CorDapp agroString application, we used Gradle. The deployNodes task in Gradle is mainly to name the nodes and configure them to a new folder. Each node has a name, port, and address. The naming convention of the Gradle uses X.500, where ‘O’ represents Organization, ‘L’ is a locality, and ‘C’ is for the country.

AgroString can take in any file format that can be uploaded and retrieved in a .zip attachment between any two nodes or parties. Node 1 sends an invoice to be received by Node 2 to download the attached .zip file to their local machine. A single state is used for the purpose of the invoice, and two flows are used for sending and downloading the files. The first flow sends and synchronizes the file between two participating logistic nodes. The files can be uploaded and attached from the local machine and can be retrieved through the linked ID. The ID generated can be used for checking if the file downloaded is the same as the file uploaded and can help for signing the attachment.

## 8. Experimental Results

### 8.1. Datasets for agroString

The datasets are collected from different sources, as shown in Table 2. These datasets are used to test and compare the current CorDapp agroString application with existing applications.

#### 8.1.1. Supply Chain Logistics Problem Data

Many internal problems arise in the supply chain, such as production hazards, improper planning, incorrect data collection, technology issues, shipping limitations, communication problems, data integrity issues, and inefficient workflows. Management assessments are being conducted for identifying the risks present in the supply chain that impede the chain and also give scope to implement an immediate possible plan. The data set provides information regarding the problems related to carrying produce between ports using different transportation modes [29].

#### 8.1.2. Livestock Farming Conditions Data

It has been claimed that livestock are raised in confined and unsanitary conditions, with little personal space and breathing toxic gases, which results in respiratory issues in the animals. The sanitation of the confined space plays an essential role in livestock’s health, hence the quality at the end. The livestock farming conditions database provides information on the geographic area where they are raised and the field’s poverty status to differentiate the livestock’s environmental setup [30].

#### 8.1.3. Fertilizer Usage in Crops

Fertilizers are mainly used to increase production and replace the nutrition that the soil loses by growing crops. Extensive usage of fertilizers can impact the environment (soil and water quality) and cause economic losses to the farmers. Excessive fertilization can impact soil and water nutrient balance and may affect crops. Fertilizer usage in the crops database gives information regarding how much fertilizer is being used for growing vegetables or other crops. With this data, the consumer can find if the amount and type of fertilizers used on the crops is within an acceptable range [31].

#### 8.1.4. Chemical Usage in Dairy

At dairy farms, the milk can have undesired ingredients due to grazing on the pesticide treated crops or because of insufficient disinfectants and detergents for cleaning operations. Most livestock farmers use veterinary drugs and other chemicals that may contain heavy metals, mycotoxins, and pesticides for efficiency in production, as well as for routine operation. These toxins and chemicals can be found in the milk, ultimately affecting the consumers [32].

#### 8.1.5. Cold Storage Data

Correct temperatures have to be maintained in every supply chain logistic stage for agricultural produce to avoid contamination, particularly in dairy and meat. Due to improper temperatures, environmental hazards and pathogen contamination can occur. The database for the cold storage has information regarding the regional and national monthly stocks of dairy, poultry, and meat products and the fruit and vegetables kept under private and semi-private refrigerated warehouses [33].

#### 8.1.6. Refrigerated Truck Volumes Data

For perishable goods (vegetables, frozen foods, ice cream, wine, etc.), different modes of transportation are being used, and trucks are one way of transferring these goods. However, for maintaining the proper hygiene of these perishable products, the transport should be refrigerated for maintaining the correct temperatures. Refrigerated trucks are becoming more popular because of their economy, moving more goods in large volumes and enhancing ease of transport over long distances. The database for refrigerated truck volume is provided by the USDA/AMS/Market News/Specialty crops program movement, which includes the information on truck mode transport and imports at the domestic origins. The data provides daily fruit and vegetable refrigerated truck volumes since 2010, truck availability for transportation, and cost for hiring these trucks [34].

#### 8.1.7. Containerized Grain Data

Containerization is a process of carrying goods in containers that are of similar shape and size. Almost any type of goods can be stored in these containers and transported through rail, road, air, and ocean modes. Ocean shipping has become more popular for loading and unloading containers, with more frequent trips and minimum lost time near the ports. The containerized grain data here gives the movements of the United States waterborne grain exports between different origins and destinations [35].

#### 8.1.8. Grain Inspection Data

Grain inspection is a process that facilitates the marketing of agricultural products, such as meat, cereals, livestock, and fishery. It gives descriptions of the product for promoting honest trading and for benefiting the consumers. The grain inspection data contains enormous volumes of information regarding United States grains inspected for export from the U.S. port regions to the destination countries. The information saved here is the data related to exported grains examined under the authority of the U.S. Grain Standards Act [36].

### 8.2. Performance Testing-Private and Public Blockchain

**Figure 9:**
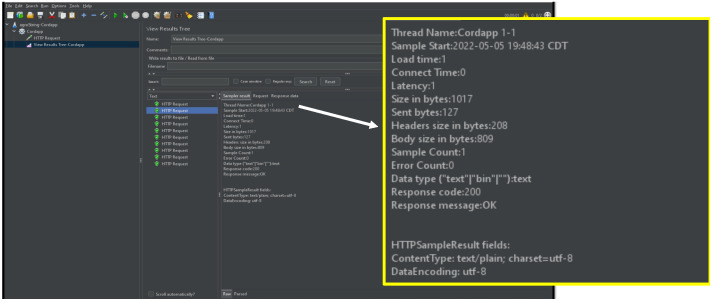
Performance Test for Corda Blockchain.

**Figure 10:**
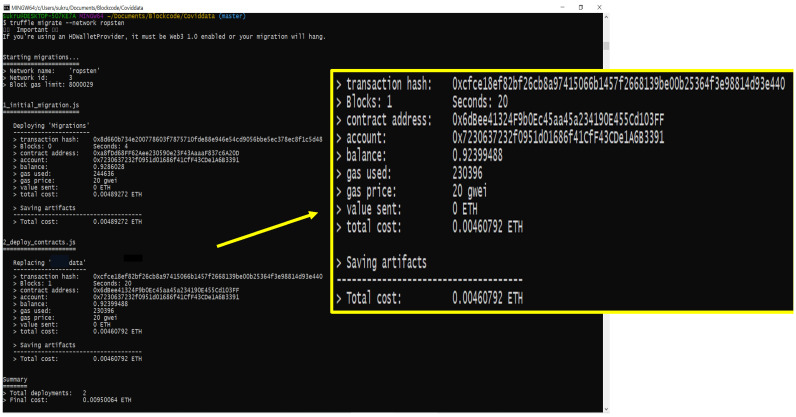
Performance Test for Public Blockchain.

JMeter Corda can be used for testing the performance of the Corda application flows. The Graphical User Interface (GUI) mode of the JMeter can be started as a client and used to create and view the test plans. The test plans can be generated by three components: Thread group, http request, and listener. We have tested agroSring dataset for private blockchain Corda, as shown in Figure 9, using JMeter and public blockchain, as given in Figure 10 through Ropsten Testnet.

Both record the load time, latency, and connecting times in milliseconds. The mining times and transaction costs are totally evaded in the Corda private blockchain, and Table 3 gives a comparison between prior works and the current agroString. The times and cost in the agro food supply chain [17] and agriculture supply chain [19] are calculated, assuming a traditional blockchain [37] to be 13.96 s [38] for 1MB Data and the cost to be $1944.84 for one Ethereum [39], respectively.

### 8.3. Why Corda Private Blockchain for agroString?

**Figure 11:**
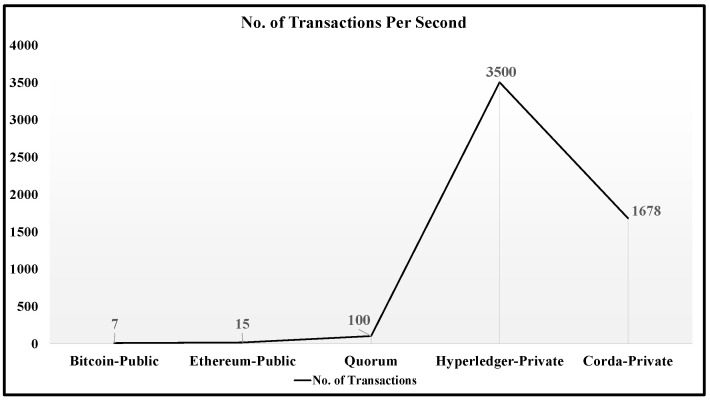
Number of transactions per second in different blockchains.

Different blockchain platforms, such as Ethereum, Hyperledger, and Corda are used for developing applications using distributed ledger technologies. Figure 11 shows a graph of transactions per second for each blockchain [41]. While all these blockchains benefit in terms of data integrity and security, they differ when it comes to vision and the field of application.

**Figure 12:**
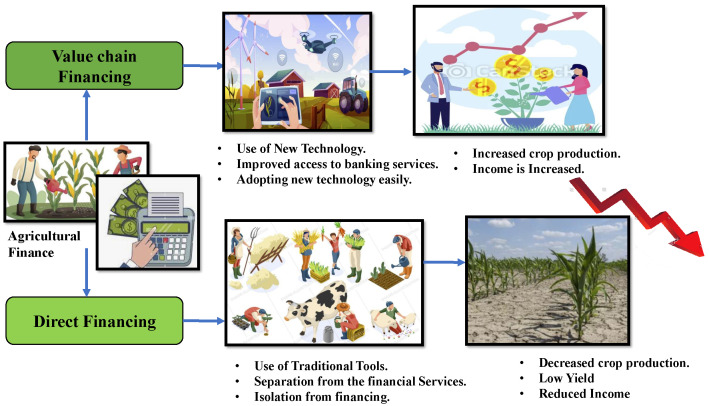
Impact of Agricultural Finance on Agricultural yield.

Both Ethereum and Hyperledger are valuable in various specific use cases, whereas Corda is most beneficial in applications related to the financial industry [42]. Producers make decisions in investment for farming mainly depending on the ease of convenience they get in accessing financial means. When the financial instruments cannot fulfill the farmer’s requirements and provide reduced-risk products, farmers are not interested in using new technologies and updated methods to increase their financial stability [43,44]. Figure 12 shows how agricultural finance influences yield in the farm sector. There are two types of financing in agriculture: *Value chain financing* is the number of steps handled by the actors or stakeholders involved in bringing the food produce to the end consumers by adding value at each stage of the product without including banks. In this financing, the actors involved provide loans and add value to the products in each logistic step. With this financing, the farmer is at an advantage in getting new types of equipment and technologies for a good yield, resulting in a reasonable price for the product and increased farmer’s economic status. *Direct* financing involves banks that give financial loans to the farmers for a certain period of repaying after the harvest.

A simple supply chain, with direct financing, moves data collected from the supplier to the consumer, but a supply chain embedded with a value chain financing concept adds value along the chain, both to the product and the stakeholders involved. Therefore, we take advantage of the Corda framework to combine the agriculture finance feature into our current agroString application.

### 8.4. Results

**Figure 13:**
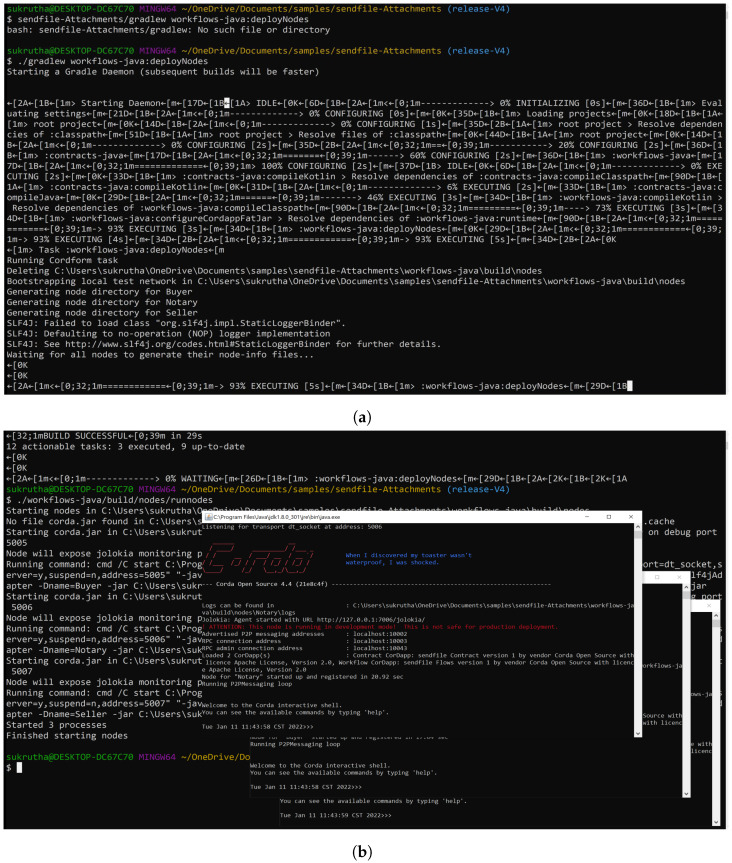

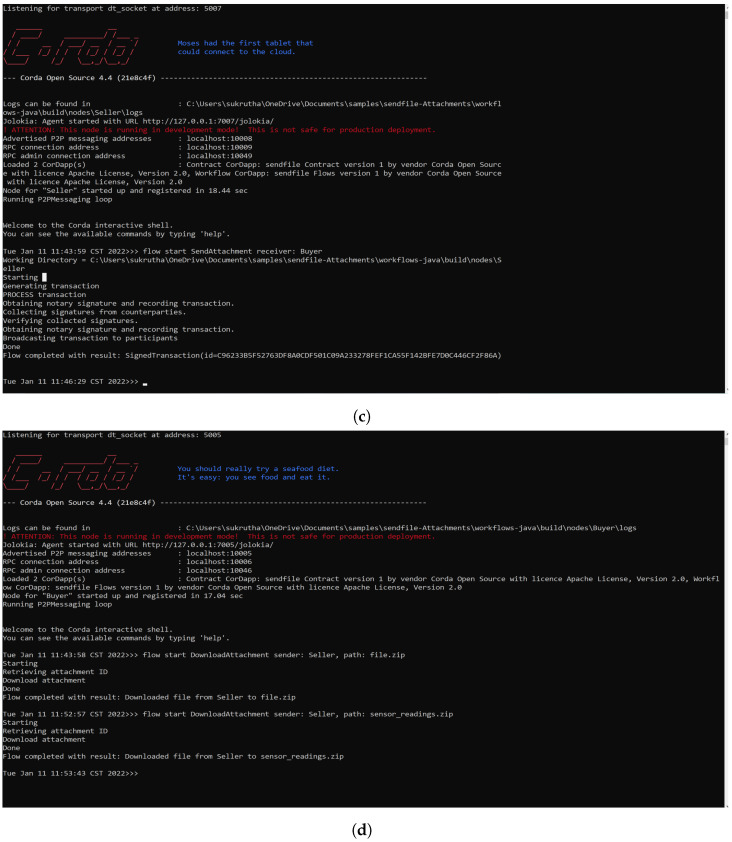
agroString CorDapp Application. (**a**) Deploying nodes with Gradle. (**b**) Running nodes. (**c**) Flows start for attachment of file. (**d**) Retrieving the file.

We deployed and ran the nodes as depicted in Figure 13a,b. Once the nodes started to run, the flow was initiated from source Node 1 to Node 2 receiver. The transaction to attach the .zip file was created and processed. Upon completing the process, the notary was used to sign and record the transaction and also obtain the counterparties’ signatures. After collecting all the signatures, they were verified, and the transaction was broadcast to the participant logistic nodes. The flow ended once the total transaction was given an ID. The entire flow is shown in Figure 13c.

The ID given from the flow start was used to check the file’s authenticity and correct origin. Here, the .zip file was attached to the receiver Node in the flow start. To retrieve the .zip file, node 2 checks for the attachment ID and verifies it to download the zip file, as shown in Figure 13d. Thus, the sensor readings and datasets were successfully transferred between relevant parties while maintaining data integrity and quality with zero transaction fees and validity times in the corDapp environment. The application takes in any file format and up to 10 MB size of the file in the form of a .zip attachment. The entire application phases are shown in Figure 13.

## 9. Conclusions and Future Direction

The agroString application is successfully built in two phases, one to generate the data from the raspberry pi and the second for storing and retrieving the generated files and datasets given in Table 2. This paper illustrates a novel architecture for the supply chain using Corda Shell and uses datasets for reading in real-time. The CorDapp agroString design is mainly based on a financial flow application that addresses the central and different blockchain system limitations. A comparative analysis of the existing approaches to the current agroString has been illustrated to show that our method gives higher data integrity and evades transactional cost and latency issues, providing more privacy and security to the data. In the future, we believe that combining both phases of IoAT into a CorDapp application can improve the agroString performance, further decreasing IoAT and blockchain challenges and enhancing security levels.

## Figures and Tables

**Table 1:** Comparison of various agricultural data applications with agroString.

Application	Data Collection	Blockchain	Cost	Storage	Security
Fish Supplychain [16]	RFID	Not used	High	Centralized	Low
agro food Supplychain [17]	RFID	Ethereum	High	Decentralized	High
Cow Tracking [18]	IoT	Not Used	High	Centralized	Low
Agriculture Supplychain [19]	IoT	Ethereum and Hyperledger	Low	Decentralized	High
Agriculture Food Supplychain [20]-Theoretical	IoT	Ethereum	Low	Decentralized	High
Traceability System [21]	IoT	Ethereum	High	Centralized and Decentralized	High
Supplychain with Blockchain [22]	IoT	Ethereum	High	Decentralized	High
Blockchain with Drones for Supplychain [24]	Drones	Ethereum	High	Decentralized	High
agroString [Current-Paper]	IoT	Corda	Low	Decentralized	High

**Table 2:** Datasets for  agroString.

Dataset Size	Data Name	Source	Link	Signed Transaction
701 KB	Supply chain logistics problem Data	Brunel University London.	Available online: https://brunel.figshare.com/articles/dataset/Supply_Chain_Logistics_Problem_Dataset/7558679/2 (accessed on 1 August 2022)	7D5F62A5141BCCFCE851C 7E1B9D974C0D0AD59B492DF D4FA20261485068694BB
516 KB	Livestock farming conditions Data	Kaggle	Available online: https://www.kaggle.com/datasets/jprukundo/ubudehelivestock1?resource=download (accessed on 1 August 2022)	01CD8FBCAC33A0A88B7D6C 1B4AF080F6EA8EDE32A90 2B2148C0694EA69571E87
12 KB	Fertilizer usage in Crops	USDA ^1^ & NASS ^2^	Available online: https://www.nass.usda.gov/Surveys/Guide_to_NASS_Surveys/Chemical_Use/ (accessed on 1 August 2022)	2B19943EA812B0D1B9 059E25D3F7F3D9CEEB94F76B C8CAE1E7620472A48DF0FE)
34 KB	Chemical usage in Diary	USDA & NASS	Available online: https://usda.library.cornell.edu/concern/publications/jh343s28d?locale=en (accessed on 1 August 2022)	020431D918FCE620E0E66D 315A808EE6552AFE23F66 2074F6F412047AFDF0375
177 KB	Cold Storage Data	USDA & NASS	Available online: https://usda.library.cornell.edu/concern/publications/pg15bd892?locale=en (accessed on 1 August 2022)	CAB13B51E194029C303E9 355BD25240E4D85B9BBAB2 6851AAE45560568CCA6D7
12.338 MB	Refrigerated Truck volumes data	USDA	Available online: https://agtransport.usda.gov/Truck/Refrigerated-Truck-Volumes/rfpn-7etz (accessed on 1 August 2022)	DF34R4632R378645D703R7 66BD65789R8F23V7GGSW5 34781AA4578678TTA4DF
406 KB	Containerized grain Data	USDA & AMS ^3^	Available online: https://agtransport.usda.gov/Container/Containerized-Grain-data/c353-2zjn (accessed on 1 August 2022)	0168135E8F56D02B6006 114BBCD8E1E3A988077E6 ACB0F42AF96D10E2D50F094
7.356 MB	Grain Inspection Data	USDA & AMS	Available online: https://agtransport.usda.gov/Exports/Grain-Inspections/sruw-w49i (accessed on 1 August 2022)	392428FA9EDA1F8D40CC2 57F10FFD1AF83B4DBF089 315F5880683DF6F4EAC1AE
15 KB	Temperature & Humidity Data	IoAT-Edge Device	IoAT-Edge Generated	4155092E577461253B2C E3FF1A9E990888536F51229 576B27FD5C06FD529EB54

^1^ U.S. Department of Agriculture. ^2^ National Agricultural Statistics Service. ^3^ Agricultural Marketing Service.

**Table 3:** Comparison of prior works with current agroString.

Application	Blockchain	Latency	Off-Chain Storage	Transaction Cost	Financial Application
Fish Supplychain [16]	RFID	Not used	High	Centralized	Low
agro food Supplychain [17]	RFID	Ethereum	High	Decentralized	High
Cow Tracking [18]	IoT	Not Used	High	Centralized	Low
Traceability System [21]	Hyperledger	0.5 s	Used-Database	Hyperledger-No Cost	No
agroString [Current-Paper]	Corda	1ms	Not Used	No Cost	Yes
1 KB = 0.032 Eth [40] 1 MB = 32.768 1 Eth = 1944.84 [38]					
